# The Food Environment in 3 Neighborhoods in South Los Angeles, California: Access, Availability, Quality, and Marketing Practices

**DOI:** 10.5888/pcd17.200028

**Published:** 2020-07-16

**Authors:** Denise D. Payán, Kathryn P. Derose, Karen R. Flórez, Cheryl A. Branch, Malcolm V. Williams

**Affiliations:** 1 Department of Public Health, School of Social Sciences, Humanities and Arts, University of California, Merced, Merced, California; 2 RAND Corporation, Santa Monica, California; 3 City University of New York, Graduate School of Public Health and Health Policy, New York, New York; 4 Los Angeles Metropolitan Churches, Los Angeles, California

**Figure:**
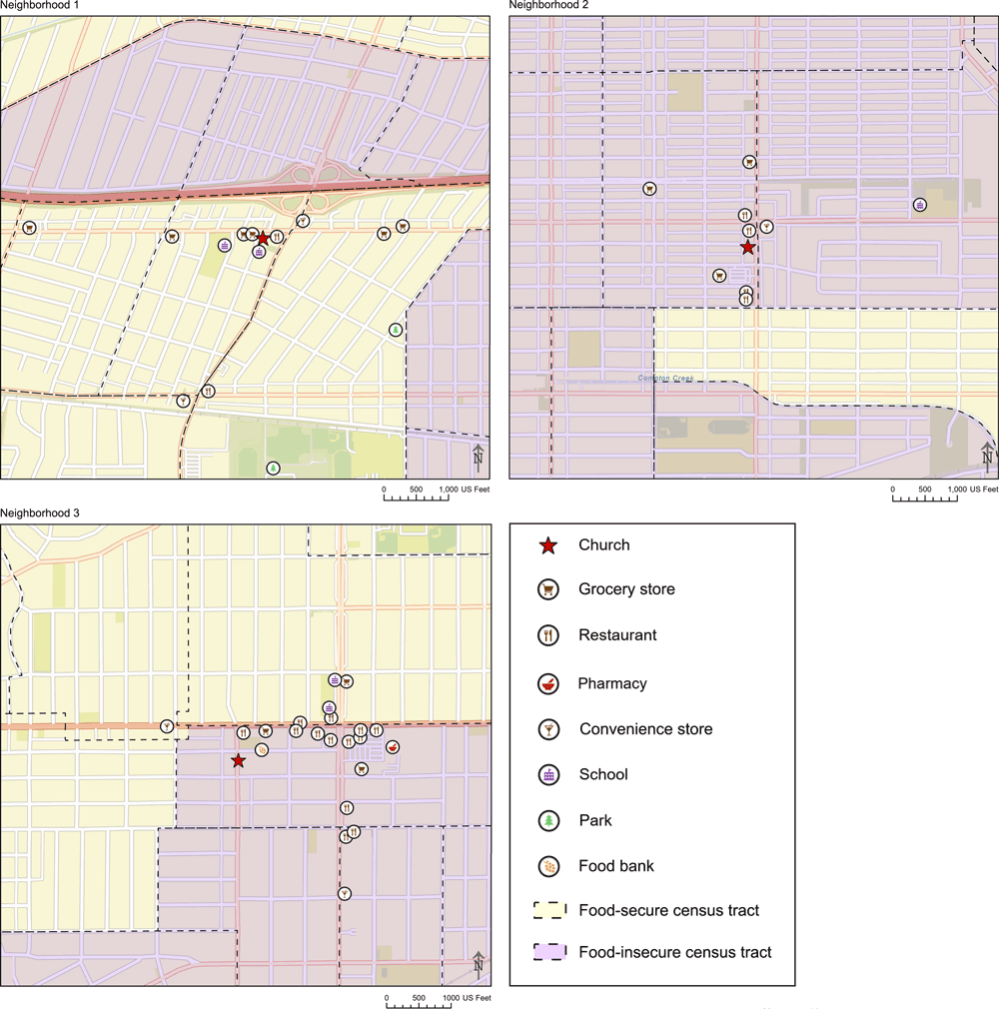
Comparison of brick-and-mortar food source types and food-insecure census tracts in 3 low-income neighborhoods in South Los Angeles, California, 2016. A food-insecure census tract was defined as a low-income census tract with ≥500 or ≥33% of residents located more than a half-mile from the nearest supermarket. Map created by Amy Newsam, SpARC Lab, University of California, Merced.

Data on the food environment can inform strategies to address obesity, particularly in food deserts, defined as low-income neighborhoods with limited access to affordable, nutritious food (1). Such data can empower residents and community-based organizations to identify policy, systems, and environmental strategies to increase access to healthy food and reduce nutrition-related health disparities in their communities (2–4).

We developed a mapping component as part of a multilevel church-based intervention that used community-based participatory research to prevent obesity in African American and Latino churches in South Los Angeles (5,6). We used the Communities of Excellence in Nutrition, Physical Activity, and Obesity Prevention (CX^3^) tools, which consist of geographic information system (GIS) mapping and field surveys to assess local nutrition and physical activity environments (3,7). We developed neighborhood maps of local food environments and provided churches with standardized information on food access, availability, quality, and marketing practices.

## Methods

Adhering to the CX^3^ GIS mapping procedures (3), we identified food sources within a half-mile radius of 3 churches (a large Roman Catholic church with mostly Latino parishioners and 2 mid-size Baptist churches with African American congregants) in South Los Angeles. Food sources were defined as grocery stores, corner stores, convenience stores, pharmacies, ethnic and specialty food stores, food service facilities, emergency food outlets, farmers markets, and mobile vendors. We used state retail data (as of August 2015) and conducted supplementary internet searches (Google, Yelp) to identify inaccuracies in commercial databases (3,4).

Two trained field workers visited all food sources, churches, parks, and schools in 3 neighborhoods (Neighborhood 1, Neighborhood 2, Neighborhood 3) in 2016 to collect data on food availability, quality of produce available in grocery stores and markets, and marketing; store food environment safety and walkability; fast food restaurants and school outdoor marketing environments; food banks and emergency food outlets; alternative food sources; and mobile vending. We observed mobile vendors near schools (ie, mobile vendors located outside of neighborhood schools) on 1 weekday after school dismissal and churches (ie, mobile vendors located outside of churches) 1 weekend after church services. Field workers also observed whether mobile vendors had an up-to-date permit visible at the point of purchase.

Field workers collected data by using printed CX^3^ forms and double-entered data into a computer spreadsheet. We calculated an index of unhealthy-to-healthy food sources for each neighborhood by dividing the number of convenience stores, fast food restaurants, supermarkets, large grocery stores, and small markets not meeting standards by the number of supermarkets, large grocery stores, and small markets meeting standards. Store addresses for brick-and-mortar food sources, schools, and parks were geocoded by using ESRI ArcGIS Pro 2.4.2 (Esri) and reviewed by a GIS researcher. Each map was overlaid with a second layer of census tract data on food insecurity: we defined food-insecure census tracts as tracts in which either ≥500 or ≥33% of residents live at least a half-mile from the nearest supermarket (1).

## Highlights

Across the 3 neighborhoods (average population, 11,724 residents), we found 37 brick-and-mortar food sources, 14 emergency and alternative food sources, 5 schools, and 2 parks (Table). The availability of healthy food varied by neighborhood. In Neighborhood 1, 58% (7 of 12) of census tracts were food secure, several supermarkets and small markets met healthy store standards, and the index of unhealthy-to-healthy food sources was 4 (8 to 2). In contrast, 50% (5 of 10) of census tracts were food secure in Neighborhood 3, and only 10% (1 of 10) of tracts were food secure in Neighborhood 2. These neighborhoods had only 1 supermarket or large grocery store each, and each neighborhood had fewer small markets and more restaurants than Neighborhood 1. The index of unhealthy-to-healthy food sources in Neighborhood 2 was 7 (7 to 1), moderately unhealthy. The unhealthiest food environment was Neighborhood 3, which had a high number of unhealthy brick-and-mortar food sources. We were unable to calculate an index in Neighborhood 3 because we found no healthy food sources.

**Table:** Availability of Food Source Types, Fresh Fruits and Vegetables, Marketing, and Outdoor Advertising in 3 Low-Income Neighborhoods in Los Angeles, California, 2016^a^

Characteristic	Neighborhood 1	Neighborhood 2	Neighborhood 3	Total
**Population**	12,470	12,464	10,239	35,173
**Population living ≤185% of the federal poverty level, no. (%)**	8,106 (65)	6,481 (52)	5,631 (55)	20,218 (57.5)
**No. of census tracts, by food-security status**				
**No. of food-insecure census tracts**	5	9	5	19
No. of food-secure census tracts	7	1	5	13
**No. of schools**	2	1	2	5
**No. of parks**	2	0	0	2
**Brick-and-mortar food sources**				
Supermarket chain or large grocery store	2	1	1	4
Small market or other market, including pharmacies	4	2	3	9
Convenience store	2	1	2	5
Fruit-and-vegetable stand	0	0	0	0
Restaurant (including fast food)	2	4	13	19
All	10	8	19	37
**Emergency and alternative food source**				
Food pantry	0	0	1	1
Mobile vendor (school)^b^	4	1	0	5
Mobile vendor (church)^c^	8	0	0	8
Farmers market	0	0	0	0
All	12	1	1	14
**Index of unhealthy-to-healthy food sources^d^**	4 (8 to 2)	7 (7 to 1)	(19 to 0)^e^	11.3
**Availability and variety of fresh fruit in food retail stores^f^**				
None	5 of 8	1 of 4	4 of 6	10 of 18
Limited (1–3 types of fruit)	0 of 8	2 of 4	1 of 6	3 of 18
Moderate (4–6 types of fruit)	0 of 8	0 of 4	0 of 6	0 of 18
Wide (≥7 types of fruit)	3 of 8	1 of 4	1 of 6	5 of 18
**Availability and variety of fresh vegetables in food retail stores^f^**				
None	5 of 8	1 of 4	4 of 6	10 of 18
Limited (1–3 types of vegetables)	0 of 8	0 of 4	0 of 6	0 of 18
Moderate (4–6 types of vegetables)	0 of 8	2 of 4	1 of 6	3 of 18
Wide (≥7 types of vegetables)	3 of 8	1 of 4	1 of 6	5 of 18
**Grocery store marketing practices^g^**				
Store meets standards for healthy marketing practices	2 of 8	1 of 4	0 of 6	3 of 18
**Restaurant marketing practices^h^**				
Restaurant meets standards for healthy marketing practices	1 of 2	1 of 4	1 of 13	3 of 19
**No. of outdoor advertisement <1,000 feet of school, park, or playground**	17	2	0	19
Presence of advertisement depicting unhealthy items or messages^i^	6 of 17	0 of 2	0	6 of 19

a State retail data (as of August 2015); internet searches (Google, Yelp), in-person data collection using the Communities of Excellence in Nutrition, Physical Activity, and Obesity Prevention (CX^3^) tools; US Department of Agriculture Economic Research Service Food Access Research Atlas, 2010-2015; US Census Bureau, 2010.

b Unhealthy food items offered by mobile vendors outside schools were defined as junk food, sugar-sweetened beverages, and ice cream/*paletas*.

c Unhealthy food items offered by mobile vendors outside church were defined as fried pork rinds/*chicharrones*, ice cream/*paletas*, bacon-wrapped hot dogs, Mexican-style corn-on-the cob, and chips.

d Index of unhealthy-to-healthy food sources was calculated as the number of convenience stores, fast food restaurants, supermarkets, large grocery stores, and small markets not meeting standards divided by the number of supermarkets, large grocery stores, and small markets meeting standards.

e Because of a lack of healthy food sources, we could not compute a score.

f Supermarket chain or large grocery store; small market or other market, including pharmacies; convenience store (n = 18).

g Collected data on marketing materials posted on the exterior (doors and windows) and interior (near check-out area) of each store.

h Collected data on marketing materials posted on the exterior and interior of each restaurant and child-oriented marketing practices (eg, photographs of unhealthy food, promotion of kids’ meal toy, availability of nutrition information). Restaurants with a marketing score ≥37 (maximum score of 50) were identified as meeting standards for healthy marketing practices.

i Includes advertisements for fast food restaurants/fast food meals or sugar-sweetened beverages.

More than half (10 of 18) of food retail stores across all 3 neighborhoods did not sell any fresh fruits or vegetables. In Neighborhood 1, three of 8 stores sold a wide variety of produce, whereas in the other neighborhoods, only 1 store offered a wide variety. Nearly all (6 of 8) brick-and-mortar stores that stocked produce had mostly higher-quality produce; 2 of 8 stores were small markets with moderate or poor-quality produce, located in Neighborhood 2 and Neighborhood 3.

Emergency and alternative healthy food outlets were scarce: we found 1 food pantry in Neighborhood 3 and no farmers market in any neighborhood. In Neighborhood 1, we observed 8 mobile vendors outside churches and 4 mobile vendors outside schools. Of the 8 mobile vendors outside the church in Neighborhood 1, four vendors primarily offered unhealthy food items, and only 1 vendor displayed a permit. All 4 mobile vendors outside schools in Neighborhood 1 and the sole mobile vendor outside a school in Neighborhood 2 offered unhealthy food items; none displayed a permit.

Only 2 of 8 grocery stores met standards for healthy marketing practices in Neighborhood 1, one of 4 grocery stores in Neighborhood 2, and 0 of 6 grocery stores in Neighborhood 3. Subway, a restaurant franchise that primarily sells submarine sandwiches, was the sole restaurant to meet healthy food standards, and each neighborhood had 1 Subway restaurant. Although we observed 17 outdoor advertisements located less than 1,000 feet of a school or park in Neighborhood 1, only 6 advertisements promoted unhealthy items or messages.

## Action

Our findings demonstrate the value of mapping food environment data at the neighborhood level to inform community-based strategies to promote healthy eating in low-income neighborhoods. The maps illustrate multiple dimensions of food insecurity — the 3 neighborhoods varied in the availability and quality of healthy food sources and items. Although 1 neighborhood (Neighborhood 1) had moderate access to healthy food, numerous mobile vendors were selling unhealthy food items near its 2 schools and the church participating in our study.

Local food environment maps that are paired with data can inform community-based strategies to prevent obesity and food insecurity. Possible strategies include corner store conversions (8) to increase fresh produce availability and reduce unhealthy food marketing (3). Examining the food environment as part of a faith-based obesity prevention project is important because churches have physical infrastructure, social networks, and other resources that could be leveraged for health promotion and advocacy. Few faith-based obesity interventions target community or policy-level strategies (9). Possible church-based strategies include developing a food pantry in a food-insecure census tract (similar to the food pantry in Neighborhood 3), distributing information on enrollment in nutrition assistance programs, collaborating with mobile food vendors to increase healthy options, and creating church-based gardens for congregants and residents.

The use of CX^3^ is a strength of our study because it includes validated instruments for assessing temporary food sources and marketing practices, which are important elements of the food environment but are often excluded from other measures (7,10) and studies (11). Additionally, we collected data on the availability and quality of several foods (4). Supplementary internet searches and in-person visits yielded a comprehensive list of food sources to identify inaccuracies in state data on retail stores (3).

Future studies could train congregants or neighborhood residents to collect and map data to promote community-driven interventions. Additional work should explore how to effectively translate mapping data into policy, systems, and environmental interventions in local contexts.
